# Green and Oolong Tea Extracts With Different Phytochemical Compositions Prevent Hypertension and Modulate the Intestinal Flora in a High-Salt Diet Fed Wistar Rats

**DOI:** 10.3389/fnut.2022.892801

**Published:** 2022-05-06

**Authors:** Xin Ye, Xiaojuan Tang, Fanglan Li, Jiangxiong Zhu, Meirong Wu, Xinlin Wei, Yuanfeng Wang

**Affiliations:** ^1^Institute of Engineering Food, College of Life Sciences, Shanghai Normal University, Shanghai, China; ^2^Department of Food Science and Technology, School of Agriculture and Biology, Shanghai Jiao Tong University, Shanghai, China

**Keywords:** green tea, oolong tea, high-salt diet, hypertension, intestinal flora

## Abstract

Green tea (GT) and oolong tea (OLT) are widely consumed beverages, and their preventive and regulatory effects on hypertension have been reported. However, the interventional effects of GT and OLT on hypertension induced by a high-salt diet and its mechanism have not been fully explored. This study evaluated the anti-hypertensive effects of GT and OLT and their underlying mechanisms. The *in vivo* anti-hypertensive effects of GT and OLT and their capability to prevent hypertension and regulate the intestinal microbiota in Wistar rats fed with a high-salt diet were evaluated. Our results show that GT and OLT supplementations could regulate oxidative stress, inflammation, gene expression, and parameter levels related to blood pressure (BP) and prevent the increase in BP induced by a high-salt diet. Furthermore, both GT and OLT boosted the richness and diversity of intestinal microbiota, increased the abundance of beneficial bacteria and reduced the abundance of harmful bacteria and conditionally pathogenic bacteria, and regulated the intestinal microbial metabolism pathway related to BP. Among them, OLT presented better effects than GT. These findings indicate that GT and OLT can prevent hypertension caused by high-salt diets, which may be due to the regulation of intestinal flora by GT and OLT.

## Introduction

High-salt intake in the western diet is an important risk factor for many cardiovascular diseases (1). It is becoming increasingly difficult to ignore the adverse effects of a high-salt diet on cardiovascular health. Emerging evidence reveals that the potential cardiovascular hazards of high-salt diets are mainly related to arterial hypertension and are significantly and positively correlated with its morbidity and mortality (2). Besides, previous intervention studies have revealed that reducing sodium salt in the diet would reduce the occurrence of cardiovascular events (3). A long-term high-salt diet can also cause other unfavorable conditions, such as the imbalance of the intestinal microecology (4). So far, most studies have focused on the direct effects on the blood vessel and kidney system (5). However, some studies have shown that gut microbes are involved in these processes, and changes in their composition and structure will affect the occurrence and development of hypertension (1).

The intestinal microorganism is an essential part of the human micro-ecological environment and crucial in maintaining human health. It is a dynamically balanced system. Intestinal microbes respond to fluctuations in the composition of the diet, resulting in transient or persistent changes in the composition of the gut microbiome (6). The intake of high-salt food can cause significant changes in the composition of the microbial community and induce hypertension, thus producing a profound impact on the host’s health (1). Accumulating evidence shows that hypertension is associated with host intestinal microflora and its metabolic disorders (7). In addition, many animal experimental models also show that hypertension causes intestinal flora imbalance (8, 9), and transplanting the dysbiological intestinal microbiota from hypertensive subjects and animal models into normotensive animals would increase the recipient’s BP (7, 10). Given the relationship between hypertension and intestinal microflora, adjusting the intestinal microflora is still a potential and effective way to reduce hypertension. The environment plays a vital role in regulating the composition and structure of intestinal microbes, especially diet (11).

Since diet has a noticeable impact on intestinal microbes, it is an executable strategy to use dietary intervention to restore the destroyed intestinal flora and ameliorate hypertension and its complications. Tea is one of the three largest non-alcoholic beverages globally, and its drinking has a history of nearly a thousand years. Tea contains many active ingredients, such as tea polyphenols, polysaccharides, proteins, and catechins, which are considered to have various health benefits (12). Many types of teas and extracts can intervene or influence the intestinal microflora and microenvironment, thus exerting its prebiotic effect (13). So far, however, there has been little discussion on tea’s impact on reducing BP by regulating the composition of intestinal microorganisms. Accumulating studies have indicated that tea has shown remarkable effects in preventing and managing hypertension (12). The epidemiological and population-based cohort results show that drinking GT or OLT can significantly reduce the risk of hypertension (14). Moreover, intervention studies of many hypertensive patients and animal models have shown that black tea and GT have a significant anti-hypertensive effect and can protect the cardiovascular system (15, 16). However, research has consistently shown that the mechanism of tea lowering BP has not been adequately investigated. To solve these issues, more related works need to be carried out.

In this work, we investigated the effects of GT and OLT on BP, metabolic disorders, and gut microbial structure and composition in high-salt-fed rats. Also, we initially explored the possible mechanism of GT and OLT to prevent hypertension. These outcomes will contribute to the development of functional hypotensive foods.

## Materials and Methods

### Materials and Reagents

Green and oolong teas were obtained from Enshi Selenium Impression Agricultural Development Co., Ltd., (Enshi, China) and Guangdong Baixiang Tea Co., Ltd., (Guangdong province, China), respectively. The cultivar of GT came from the local area of Enshi, named ‘Enshi Taizi’, contained four or five leaves with fully mature. The cultivar of OLT was semi-treerescent form and sexual group and it contained one bud and four or five leaves with fully mature. Glutathione peroxidase (GPX), superoxide dismutase (SOD), malondialdehyde (MDA), nitric oxide (NO), creatinine (Cre), aldosterone (ALD), angiotensin-converting enzyme II (Ang II), and c-reactive protein (CRP) detection kits were purchased from Nanjing Jiancheng Bioengineering Institute (Nanjing, China). All catechin standards used in liquid chromatography were purchased from Chengdu RefMedic Biotech Co., Ltd., (Chengdu, China). All 21 amino acid standards were purchased from Sigma Co., (St Louis, MO, United States). All other chemicals were of analytical grade unless otherwise specified.

### Tea Aqueous Extract Preparation

Tea leaves were crushed and then extracted (1:10 and 1:9, w/v) twice with boiling water for 4 h. After filtering through 500 mesh nylon cloth, the extracts were combined and centrifuged, and then the supernatant was concentrated and lyophilized.

### Chemical Profile Analysis

The polysaccharide content was measured by the phenol sulfuric acid method (17). The polyphenol content was determined by using Folin–Ciocalteu method regarding the national standard of China (GBT8313-2018). The flavonoid content was investigated according to the description of the national standard of China (SNT4592-2016). The element distribution was percormed according to the previous report (18). The catechin, alkaloid, and phenolic acid contents were measured according to the previous report by our lab (19). A high-performance liquid chromatography LCC-AT20 system (Shimadzu, Tokyo, Japan) was used to analyze the amino acid content in the samples. All kinds of amino acid standards (dissolved in 0.1 mol/L HCl) were prepared into a 1 mg/mL solution, and then each standard solution was mixed equally and diluted to each concentration gradient with 0.1 mol/L HCl. The samples were prepared in the same way. A total of 200 μL combined standard solution or sample was dissolved in mixed solution (200 μL OPA derivatization reagent + 600 μL boric acid buffer) and derivatized for 15 min in the dark. Next, HPLC analysis was performed. The HPLC system was as follows: C18 column (250 mm × 4.6 mm, 5 μm), 35°C for column temperature, 1 mL/min for flow rate, 338 nm for detection wavelength, 20 μL for injection, mobile phase A: 20 mmol/L dihydrogen phosphate sodium solution; mobile phase B: a mixed solution (methanol: acetonitrile: distilled water = 45: 45: 10).

### Animals and Experimental Design

Twenty-four 8-week-old cleaning Wistar male rats were obtained from Slaccas Laboratory Animal Co., Ltd., (Shanghai, China) and divided into four groups, including model control (MC), GT, OLT, and normal control (NC) groups. All animal experiments were carried out according to the Experimental Animal Ethics Standards of the Experimental Animal Ethics and Use Committee of Shanghai Jiao Tong University (approval A2020080) and the Laboratory animal-Guideline for ethical review of China to maximize animal welfare. All experimental rats were housed at the Animal Experiment Center of Shanghai Jiao Tong University with free access to food and water in a controlled animal room (25 ± 1°C, 70–75% humidity, and a 12 h light-dark cycle). After acclimatization for 1 week, the NC group received Shoobree common standard feed (No. 1010009, Jiangsu Synergy Pharmaceutical Bioengineering Co., Lt d., Jiangsu, China) for 9 weeks on a regular diet; MC, GT, and OLT groups received with high-salt chow (92.45% common standard feed + 7.55% sodium chloride, 20210308(x), Suzhou Hongxin Biotechnology Co., Ltd., Jiangsu, China) for 9 weeks to induce hypertension. The Shoobree typical standard feed composition was presented in our previous report (20). In addition, the rats in GT and OLT groups were given 500 mg/kg GT and OLT aqueous extracts added into drinking water daily, respectively, and the rats in MC and NC groups were given distilled water. At the start and end of treatment, the body weight and systolic pressure reflecting BP of rats were measured.

### Histology Analysis

After dehydration, the heart and kidney tissues were embedded in paraffin and sliced (3 μm of thickness) and then placed in an oven at 60°C for 30–60 min. Next, gradient staining was performed according to the following steps: xylene I for 5 min, xylene II for 5 min, xylene III for 5 min, absolute ethanol for 1 min, 95% alcohol for 1 min, and 75% alcohol for 1 min. After washing, the sections were stained with hematoxylin staining solution for 2 min. After washing and returning to blue, the gradient dyeing was continued according to the following steps: eosin staining solution for 1 min, 75% alcohol for 30 s, 95% alcohol for 30 s, absolute alcohol for 30 s, xylene transparent for 1 min. Finally, the sections were mounted, dried, and observed under an optical microscope (× 400).

### Real-Time Reverse Transcription-Quantitative PCR (qRT-PCR) Analysis

The total mRNA in kidney tissue was extracted and reverse transcribed into cDNA according to the Servicebio^®^RT First Strand cDNA Synthesis Kit instructions (Service, Wuhan, China). The mRNA expression level was detected by SYBR qPCR Master Mix (High ROX, Wuhan, China) according to the light quantitative PCR kit instructions. The specific primers used were as follows: ACE, 5′-TCATCCAGTTCCAGTTCCACG-3′ (F), 5′-CGTGTTTGGTGTCCAGG-3′(R); endothelin-1 (ET-1), 5′-TTGCTCCTGCTCCTCCTTGAT-3′(F), 5′-CTGTTCCCTTGG TCTGTGGTC-3′(R); endothelial nitric oxide synthase (eNOS), 5′-GGTATTTGATGCTCGGGACTGC-3′(F), 5′- GTGATGG CTGAACGAAGATTGC-3′ (R). β-actin, 5′-TGCTATGTTGC CCTAGACTTCG-3′ (F), 5′-GTTGGCATAGAGGTCTTTAC GG-3′ (R). The gene β-actin was employed as an internal reference, and the relative mRNA level of target genes was calculated using the 2−ΔΔCt method.

### Biochemical Analysis

The blood of rats was collected by cardiac puncture. Then, blood was immediately managed and centrifuged at high speed (10,000 rpm) at 4°C for 10 min. Serum was collected and stored at −80°C. The levels of GPX, SOD, MDA, NO, Cre, ALD, Ang II, and CRP in serum were measured by commercially available kits.

### Microbiome Profiling of Fecal Samples

The tail of the fixed rats was lifted, and the lower abdomen of the rats was gently pressed. After that, fresh feces were collected aseptically and stored at −80°C until detection. Detailed DNA extraction analysis and sequencing steps were summarized in Supplementary Information.

### Statistical Analysis

The data were expressed as the arithmetic mean ± standard deviation. The student’s *t*-test assessed comparisons between groups for two groups and one-way ANOVA for multiple groups using the Tukey test. A level of *p* < 0.05 was considered statistically significant. All statistical analysis was performed by SPSS 20.0 (SPSS Inc., Chicago, IL, United States) and GraphPad Prism 8.0 (GraphPad Software Inc., San Diego, CA, United States).

## Results

### Chemical Composition

As shown in Table 1 and Supplementary Figure 1, both GT and OLT extracts contained appreciable contents of tea polysaccharides, polyphenols, epigallocatechin gallate (EGCG), epigallocatechin (EGC), total flavonoids, and L-theanine. Therein, the higher contents of EGCG, EGC, and L-theanine were found in OLT extracts, and the higher contents of tea polysaccharides, polyphenols, and total flavonoids were found in GT extracts. In addition, the two tea extracts also contained a high content of free amino acids, alkaloids and element distributions (Supplementary Table 1). However, most of the components of the two tea extracts had significant differences, which might be related to their sources and processing techniques. These results indicate that both GT and OLT extracts contain beneficial nutrients, which may be the main contributors to the regulation of BP.

**TABLE 1:** Comparison of GT and OLT extracts for the main components (*n* = 3).

Taxonomy	Category	GT	OLT
Polysaccharides (mg/g)	Tea polysaccharides	283.02 ± 7.09	176.23 ± 5.14**
Tea polyphenols (mg/g)	Total polyphenols	221.30 ± 1.82	192.49 ± 1.12**
Flavone (mg/g)	Total flavonoids	30.61 ± 0.63	20.49 ± 0.11**
Catechins (mg/g)	Catechin	5.31 ± 0.07	11.07 ± 0.02**
	EC	16.29 ± 0.39	9.00 ± 1.42**
	ECG	12.06 ± 0.19	11.18 ± 0.20**
	GC	48.36 ± 0.85	8.62 ± 0.35**
	EGC	52.46 ± 0.98	59.36 ± 2.80*
	GCG	18.24 ± 0.30	10.24 ± 0.53**
	EGCG	57.24 ± 0.98	86.31 ± 0.75**
Alkaloids (mg/g)	Caffeine	62.69 ± 0.35	8.40 ± 0.59**
	Theobromine	13.50 ± 0.23	21.64 ± 0.07**
Phenolic acids (mg/g)	Gallic acid	3.78 ± 0.05	20.05 ± 0.13**
	Caffeic acid	0.81 ± 0.01	0.83 ± 0.09ns
	p-Coumaric acid	1.40 ± 0.02	1.24 ± 0.05*
	Ferulic acid	0.59 ± 0.01	0.69 ± 0.03*
Amino acids (mg/g)	Asp	6.72 ± 0.05	3.38 ± 0.02**
	Glu	6.50 ± 0.17	0.65 ± 0.07**
	Asn	1.99 ± 0.01	0.87 ± 0.01**
	Gln	0.35 ± 0.01	0.62 ± 0.01**
	Gly	0.35 ± 0.01	0.65 ± 0.32**
	His	0.44 ± 0.02	0.51 ± 0.04*
	Thr	1.27 ± 0.05	1.04 ± 0.09**
	Pro	0.15 ± 0.01	0.15 ± 0.07ns
	Ala	1.14 ± 0.01	0.58 ± 0.04**
	Ser	0.36 ± 0.05	1.34 ± 0.08**
	L-theanine	20.68 ± 0.02	21.53 ± 0.03*
	Tyr	0.08 ± 0.01	3.51 ± 0.07**
	Arg	2.79 ± 0.12	2.12 ± 0.18ns
	Val	0.17 ± 0.01	0.36 ± 0.02**
	Met	1.40 ± 0.13	1.65 ± 0.15**
	Trp	1.95 ± 0.13	2.61 ± 0.15**
	Ile	0.78 ± 0.05	2.47 ± 0.30**
	Phe	0.21 ± 0.03	0.44 ± 0.02**
	Leu	0.70 ± 0.07	0.64 ± 0.12ns
	Lys	1.75 ± 0.13	4.27 ± 0.33**
	Cys	ND	ND

*ND, not detected. Asterisk indicates a significant difference compared to GT.*

*p < 0.05, **p < 0.01. EC, epicatechin; ECG, epicatechin gallate; GC, gallocatechin; EGC, epigallocatechin; GCG, gallocatechin gallate; EGCG, epigallocatechin gallate.

### Effect of Green Tea and Oolong Tea on Body Weight, Blood Pressure, and Histology of the Heart and Kidney

**FIGURE 1:**
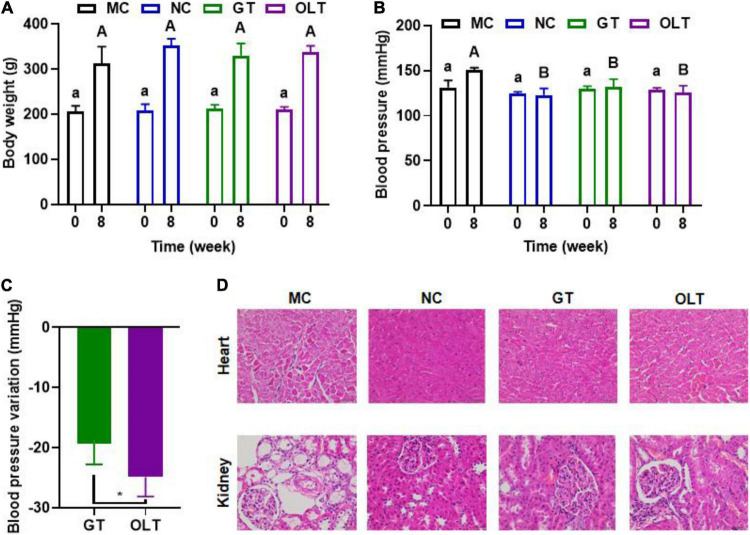
GT and OLT affected the body weight, blood pressure, and tissue condition. **(A)** Body weight. **(B)** Blood pressure. **(C)** Blood pressure variation after GT and OLT supplementation, **(D)** Histological analysis of heart and kidneys. Different uppercase or lowercase letters represent a significant difference among multiple groups (*p* < 0.05). Asterisk represent a significant difference between groups. *p < 0.05. One-way ANOVA analysis followed by a Tukey test was employed to estimate the statistical significance.

To assess the implication of a high-salt diet on the body weight and BP of Wistar rats before and after drinking tea were measured. Overall, a long-term high-salt diet did not significantly affect the body weight, and the intervention of teas had limited effects on the body weight (Figure 1A). However, the treatment of GT or OLT prevented the increase in BP caused by a high-salt diet, and the BP of the GT and OLT groups was significantly different from that of the MC group after 8 weeks of intervention (Figure 1B). Moreover, the effect of OLT on preventing the increase of BP was better than GT (Figure 1C). Further histological analysis shows that both GT and OLT reversed the structural damage of the heart and kidney tissue caused by a high-salt diet (Figure 1D), including hypertrophy and necrosis of cardiomyocytes, thickening of the arterial walls of small blood vessels in the myocardium, and glomerular capillary dilation, as well as vacuolization, degeneration and necrosis of renal tubular epithelial cells.

### Effect of Green Tea and Oolong Tea on the Gene Expression

**FIGURE 2:**
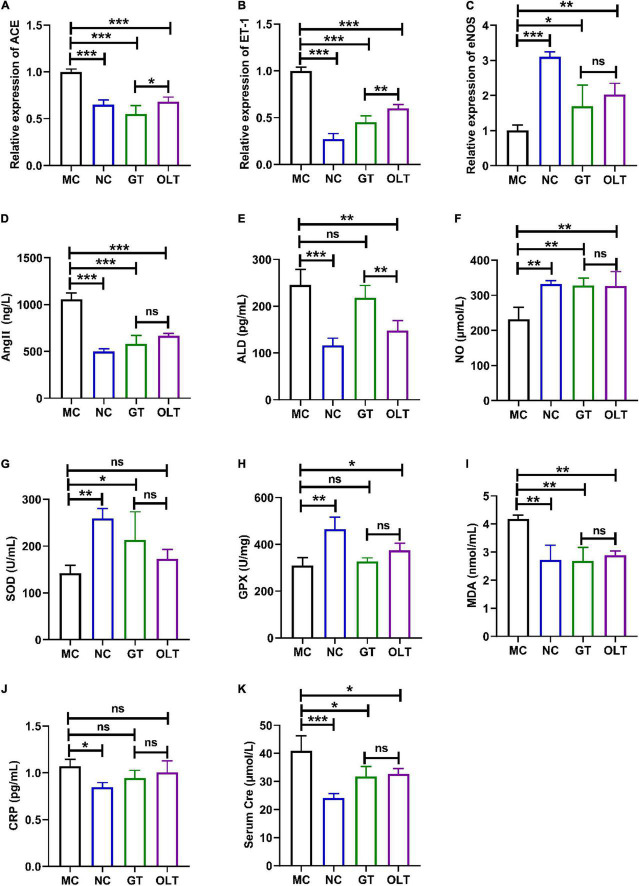
GT and OLT adjusted the gene expression related to blood pressure and the serum biochemical parameters related to blood pressure in rats fed a high-salt diet. The relative expression of ACE **(A)**, ET-1 **(B)**, and eNOS **(C)**. The levels of Ang II **(D)**, ALD **(E)**, NO **(F)**, SOD **(G)**, GPX **(H)**, MDA **(I)**, CRP **(J)**, and Cre **(K)**. Asterisk represent a significant difference between groups. *p < 0.05; **p < 0.01; ***p < 0.001. One-way ANOVA analysis followed by a Tukey test was employed to estimate the statistical significance.

Studies have shown that a long-term high-salt diet causes an increase in BP (1). Thus, we investigated the effect of GT and OLT on the gene expressions closely related to BP regulation in the kidneys, including ACE, ET-1, and eNOS. From the data in Figures 2A–C, compared with the NC group, the mRNA expression of ACE and ET-1 of the MC group increased significantly, while the eNOS expression decreased significantly. However, compared with the MC group, GT and OLT treatments significantly down-regulated the mRNA expressions of ACE and ET-1 and significantly up-regulated the mRNA expression of eNOS. In particular, OLT exhibited a stronger regulatory effect than GT.

### Effect of Green Tea and Oolong Tea on the Serum Biochemical Parameters

Ang II, ALD, and NO are important regulators to maintain the balance of BP in the body, and their aberrant level will have a meaningful impact on BP (21, 22). As shown in Figures 2D–F, the MC group reported significantly more Ang II and ALD levels and lowered NO level than the NC groups. GT or OLT administration remarkably reduced the Ang II and ALD levels and considerably elevated the NO level. Therein, only a limited regulation by GT on the ALD level was presented. Besides, the regulation effect of OLT on ALD was also better than GT.

The continuous increase of BP will increase the degree of oxidative stress and inflammation, which will lead to vascular dysfunction and kidney damage (23). Figures 2G–J shows a significant decrease in SOD and GPX enzyme activities and a significant increase in MDA and CRP levels in the MC group. For SOD and GPX enzyme activities, GT and OLT treatments significantly enhanced the enzyme activities of SOD and GPX, respectively. GT and OLT treatments noticeably decreased the MDA level but had a limited effect on CRP level for MDA and CRP levels. CRE is one of the markers of renal injury, and damaged kidneys are usually accompanied by elevated serum CRE levels (24). As shown in Figure 2K, a long-term high-salt diet caused a significant increase in the Cre level in the MC group, but this could be significantly reversed by GT or OLT intervention. Interestingly, GT and OLT showed similar effects on the regulation of oxidative stress, inflammation, and kidney damage (no significant difference between groups).

### Effect of Green Tea and Oolong Tea on the Diversity of Intestinal Flora

**FIGURE 3:**
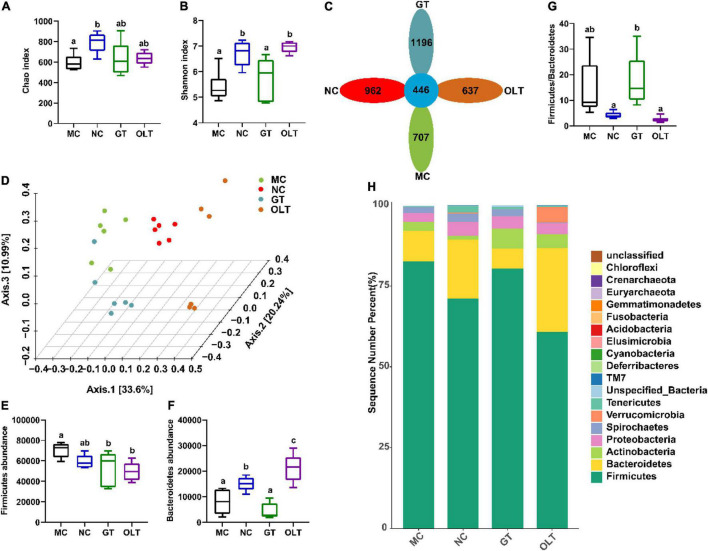
GT and OLT altered the gut microbial diversity and composition at the phylum level in rats induced by a high-salt diet. **(A)** Chao index reflects species richness (the number of species). **(B)** Shannon index assessing the species diversity. **(C)** Flower diagram of OTUs, the petals are the number of OTUs unique to the corresponding group, and the center is the number of mutual OTUs of all groups. **(D)** 3D PCoA analysis based on Bray Curtis distance, the percentage represents the contribution value of each principal component to the sample difference. **(E)** Firmicutes abundance at the phylum level. **(F)** Bacteroidetes abundance at the phylum level. **(G)** The ratio of Firmicutes to Bacteroidetes. **(H)** Histogram of the relative distribution of gut microbes at the phylum level. One-way ANOVA analysis followed by a Tukey test was employed to estimate the statistical significance. The different letters represent significant differences between different groups (*p* < 0.05).

16S rRNA high-throughput sequencing technology was used to sequence the microbiota in fecal samples of rats on the Illumina novaseq platform. A total of 2,322,739 valid sequences and 4,801 different OTUs were provided from 24 samples (*n* = 6 in each group). OTU and Shannon rarefaction curves (Supplementary Figure 2) indicate that the number of sequencing samples is sufficient, and the species richness and community uniformity are both high. The results obtained from the preliminary analysis of α-diversity, including the Chao and Shannon indices, are presented in Figures 3A,B. As can be seen, the high-salt diet caused a significant decrease in the OTU richness and community α-diversity of the intestinal flora. At the same time, the intervention of GT and OLT alleviated the decline in the OTU richness and α-diversity caused by the long-term high-salt diet. Among them, OLT showed a better alteration effect. The flower diagram of OTUs (Figure 3C) presented 446 mutual OTUs of all groups. The peculiar OTUs in MC, NC, GT, and OLT groups were 707, 962, 1,196, and 637. PCoA analysis (β-diversity) (Figure 3D) showed a complete separation of gut microbiota community between the MC and NC groups. The intervention of GT and OLT reshaped the gut microbiota destroyed by the high-salt diet and brought it close to a healthy state, particularly OLT, with more effectiveness.

### Effect of Green Tea and Oolong Tea on the Gut Microbiota Composition at the Phylum Level

At the phylum level (Figure 3H), the intestinal flora structure of rats was dominated by Firmicutes and Bacteroidetes. Compared with the NC group, the continuous high-salt diet feeding caused a significant increase in Firmicutes and a significant decrease in Bacteroidetes in the intestinal microbes of the MC group (Figures 3E,F). As a result, an elevated ratio of Firmicutes to Bacteroides in the MC group was found (Figure 3G). Conversely, OLT supplementation significantly decreased Firmicutes and significantly increased Bacteroidetes. Accordingly, OLT supplementation could substantially reduce the ratio of Firmicutes to Bacteroides, while GT supplementation had a limited effect.

### Effect of Green and Oolong Teas on the Gut Microbiota Composition at the Genus Level

**FIGURE 4:**
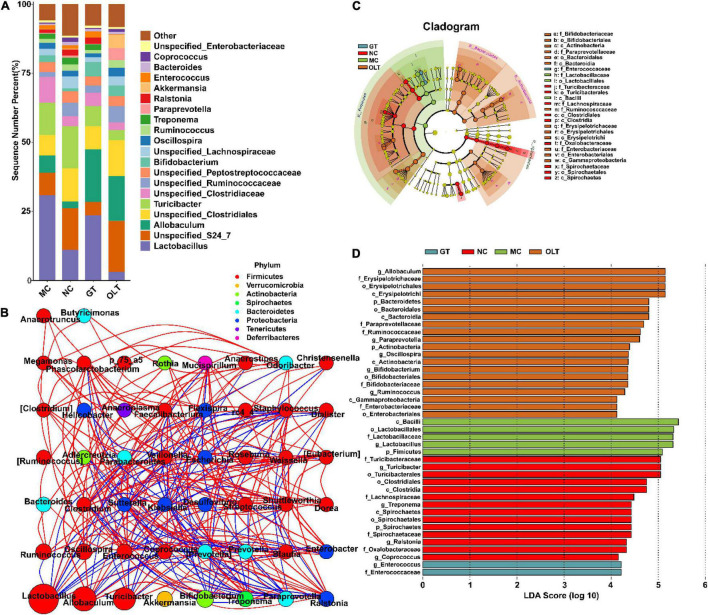
GT and OLT changed gut microbiota structure at the genus level in rats in response to a high-salt diet. **(A)** Histogram of the relative distribution of gut microbes at the genus level. **(B)** Microbial interaction network diagram at the genus level based on Spearman correlation analysis. The circle represents a genus, the size represents its relative abundance, different colors represent different phylum classifications, the line between the circles represents a significant correlation between the two bacteria (*p* < 0.05). The red line represents a positive correlation, and the blue line represents a negative correlation. The thicker the line, the greater the absolute value of the correlation coefficient. **(C)** Cladogram based on LEfSe analysis. The cladogram corresponds to different levels of intestinal microbial classification from the inside to the outside, and the connection between the levels represents the belonging relationship. Each circled node represents a classification of bacteria, yellow nodes represent insignificant differences between groups, and non-yellow nodes represent that the bacterium is characteristic microorganisms of the corresponding group (significantly higher abundance in this group). The colored fan-shaped area marks the subordinate classification intervals of the characteristic microorganisms. **(D)** LDA histogram based on LEfSe analysis. Each horizontal column represents a kind of bacterium, and the length of the column corresponds to the LDA value. The higher the LDA value, the greater the difference. The color of the column corresponds to the characteristic microorganisms of the bacterial group (the higher abundance in the corresponding group).

The intestinal microbes (top 20 genera in relative abundance) at the genus level in different treatment groups also had obvious distinctions (Figure 4A). Additionally, Spearman correlation analysis based on the relative abundance showed more or less antagonistic or synergistic effects among various bacteria in rats of each group (Figure 4B). The cladogram in Figure 4C and linear discriminant analysis (LDA) histogram in Figure 4D indicate that the GT group specifically and significantly enriched the *Enterococcus* genus compared with other groups. In contrast, the OLT group was characterized by specific and significant enrichment for the *Allobaculum*, *Paraprevotella*, *Oscillospira*, *Bifidobacterium*, and *Ruminococcus* genera. Likewise, a significant selective enhancement for *Turicibacter*, *Treponema*, *Ralstonia*, and *Coprococcus* genera in the NC group was found. However, the most unexpected result from this data is that the MC group specifically and significantly enriched the abundance of *Lactobacillus*.

### Difference in Composition and Enrichment of Bacteria Genera With High Abundance

**FIGURE 5:**
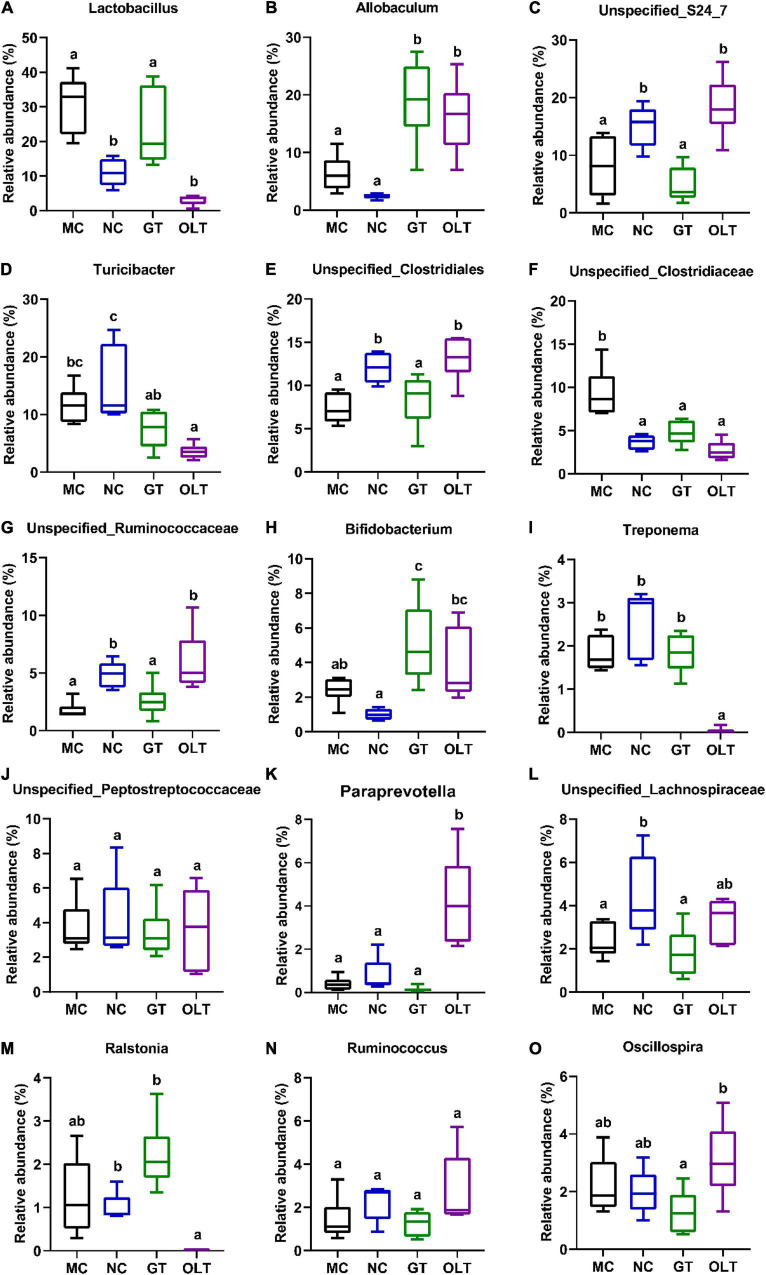
The relative abundance of top 15 genera in gut microbial of high-salt fed rats differed after GT and OLT supplementation. **(A)** Lactobacillus, **(B)** Allobaculum, **(C)** Unspecified_S24_7, **(D)** Turicibacter, **(E)** Unspecified_Clostridiales, **(F)** Unspecified_Clostridiaceae, **(G)** Unspecified_Ruminococcaceae, **(H)** Bifidobacterium, **(I)** Treponema, **(J)** Unspecified_Lachnospiraceae, **(K)** Paraprevotella, **(L)** Unspecified_Lachnospiraceae, **(M)** Ralstonia, **(N)** Ruminococcus, **(O)** Oscillospira. One-way ANOVA analysis followed by a Tukey test was employed to estimate the statistical significance. The different letters represent significant differences between different groups (*p* < 0.05).

Just like the ecosystem, not all species are equal. The number of main microorganisms in the intestinal micro-ecosystem may have a crucial impact on the intestinal microenvironment. As shown in Figure 5, the top 15 genera (more than 98% abundance) had mainly consisted of *Lactobacillus*, *Allobaculum*, *Unspecified_S24_7*, *Turicibacter*, *Unspecified_Clostridiales*, *Unspecified_Clostridiaceae*, *Unspecified_Ruminococcaceae*, *Bifidobacterium*, *Treponema*, *Unspecified_Lachnospiraceae*, *Paraprevotella*, *Unspecified_Lachnospiraceae*, *Ralstonia*, *Ruminococcus*, and *Oscillospira*. In high-salt fed rats, several bacteria were significantly decreased compared with a regular diet, including *Unspecified_S24_7*, *Turicibacter*, *Unspecified_Clostridiales*, *Unspecified_Ruminococcaceae*, *Unspecified_Lachnospiraceae*, and *Ralstonia* whereas others were significantly increased, such as *Lactobacillus* and *Unspecified_Clostridiaceae*. Interestingly, the decreased abundance of *Unspecified_S24_7*, *Unspecified_Clostridiales*, *Unspecified_Ruminococcaceae*, and *Unspecified_Lachnospiraceae* observed in the MC group was remarkably reversed by OLT supplementation. Furthermore, OLT intervention significantly reversed the high-salt evoked increase in the abundance of *Unspecified_Clostridiaceae*. Accordingly, GT intervention significantly elevated the relative abundance of *Ralstonia* and notably reduced the relative abundance of *Unspecified_Clostridiaceae*. Of particular interest, GT and OLT supplementation also considerably increased the relative abundance of *Allobaculum* and *Bifidobacterium*. However, surprisingly, the MC group was characterized by a relatively higher abundance of *Lactobacillus* traditionally classified as beneficial microbe.

### Gut Microbial OTU Composition and Its Correlation With Metabolic Parameters

**FIGURE 6:**
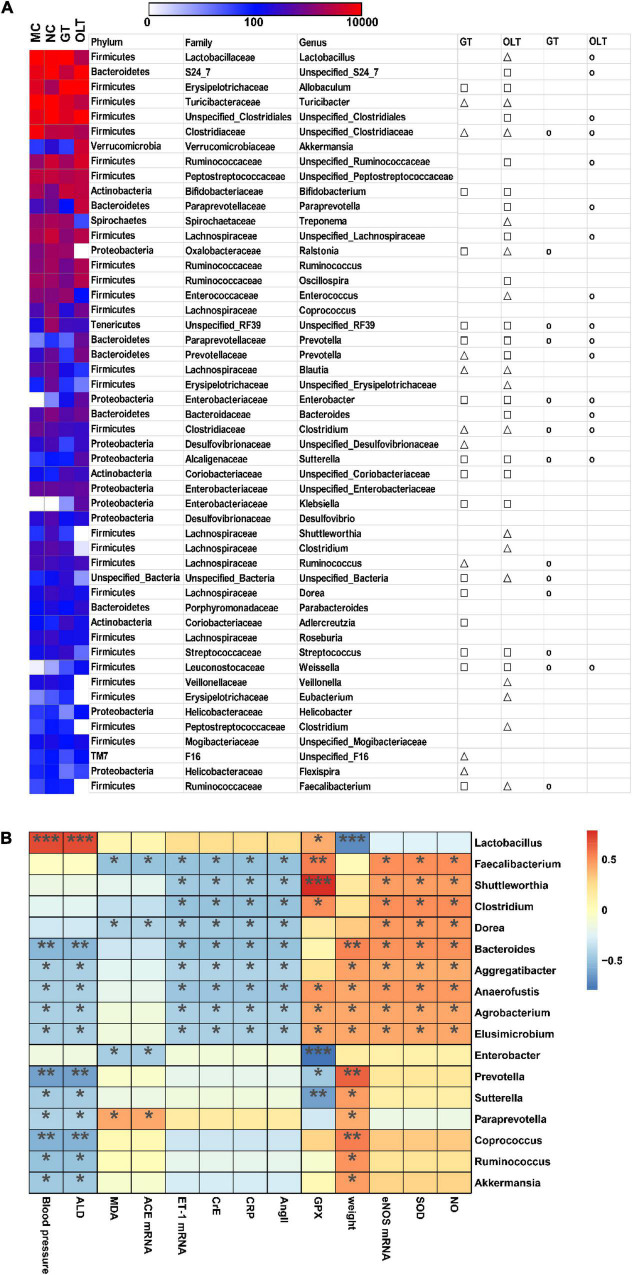
GT or OLT treatment reversed the imbalance of intestinal flora abundance of rats caused by a high-salt diet. **(A)** Heatmap in the abundance of the top 50 OTUs of different treatment groups of high-salt fed rats. The triangle and square indicate the less and more relative abundances of OTUs in GT or OLT groups in comparison with the MC group, respectively. The circular indicates that the OTU of the NC group that was changed by hypertension was reversed by GT or OLT treatment. **(B)** Spearman correlation analysis between the microbial genera in the intestinal flora and related parameters of hypertension. The parameters related to blood pressure are on the X-axis, and the bacteria genera are on the Y-axis. The R-value is displayed in different colors, and the * sign indicates that there is a significant correlation between the two. *p < 0.05, **p < 0.01, ***p < 0.001.

Among the top 50 OTUs (more than 99% abundance), 24 distinct OTUs had undergone noticeable variations through the GT intervention, and 34 distinct OTUs were significantly changed by OLT intervention compared with the MC group (Figure 6A). Further analysis revealed that 13 and 16 of the OTUs altered by the Control group were reversed in response to GT and OLT interventions, respectively. Likewise, Spearman correlation analysis (Figure 6B) was employed to investigate the correlation between the top 50 OTUs and hypertension-associated metabolic parameters. As can be seen, 17 out of 50 OTUs were positively or negatively correlated with at least one parameter associated with hypertension. Therein, *Bacteroides*, *Aggregatibacter*, *Anaerofustis*, *Agrobacterium*, *Elusimicrobium*, *Prevotella*, *Sutterella*, *Paraprevotella*, *Coprococcus*, *Ruminococcus*, and *Akkermansia* were negatively and significantly correlated with hypertension. However, although *Lactobacillus* had a significant correlation with hypertension but showed a controversial effect. Also, *Faecalibacterium*, *Shuttleworthia*, *Clostridium*, *Dorea*, *Bacteroides*, *Aggregatibacter*, *Anaerofustis*, *Agrobacterium*, *Elusimicrobium*, *Enterobacter* and *Paraprevotella* were negatively and significantly correlated with hypertension-related disorders. These genera may play a key role in preventing hypertension evoked by a high-salt diet.

### Green Tea and Oolong Tea Regulated the KEGG L3 Pathways Related to Hypertension

**FIGURE 7:**
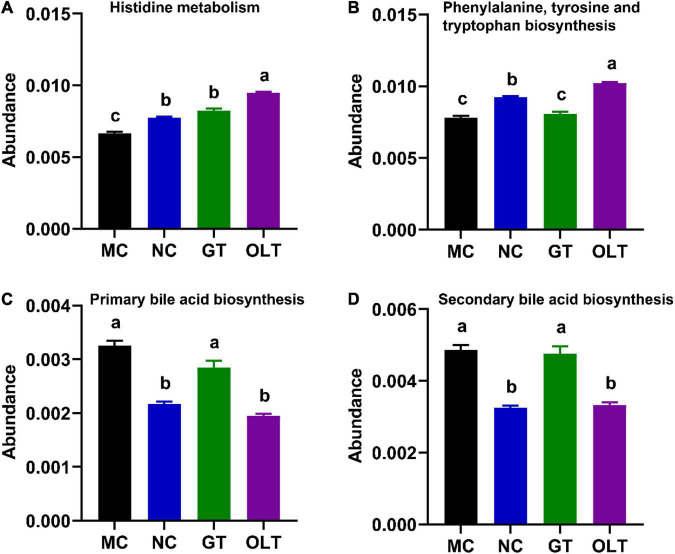
GT and OLT regulated the KEGG L3 pathway related to hypertension. **(A)** Histidine metabolism, **(B)** Phenylalanine, tyrosine, and tryptophan biosynthesis, **(C)** Primary bile acid biosynthesis, **(D)** Secondary bile acid biosynthesis. If and only if the *p*-value after ANOVA analysis corrected for “false discovery rate” is less than 0.05, the Duncan test will be further performed. The different letters represent significant differences between different groups (*p* < 0.05).

Up to date, identified pathways of particular interest regarding BP regulation, including histidine metabolism, tryptophan metabolism, and bile acid metabolism, have been proved to affect host BP through circulating microbial metabolites (11). Based on these researches, the KEGG L3 pathways related to hypertension, including histidine metabolism, phenylalanine, tyrosine, and tryptophan biosynthesis, primary bile acid biosynthesis, and secondary bile acid biosynthesis, were investigated to explore the effect of GT or OLT intervention on intestinal microbial function (Figure 7). OLT supplementation significantly enhanced histidine metabolism and phenylalanine, tyrosine, and tryptophan biosynthesis compared with the MC group. Moreover, it also significantly decreased the primary and secondary bile acid biosynthesis. However, GT exhibited a limited impact on these pathways.

## Discussion

A western high-salt diet is a risk factor for cardiovascular complications and metabolic syndromes (25). Despite its indispensable involvement in many physiological activities, excessive salt uptake is detrimental to many well-recognized diseases, especially hypertension (25). Changing dietary habits has been proven to regulate BP (25) effectively. In particular, nutritional therapy has exhibited beneficial effects on the prevention and management of hypertension. As mentioned in the literature review, a strong relationship between tea consumption and BP has been reported in animal, population-based cohort, and meta-analysis studies (26, 27). Moreover, prior studies have noted the beneficial effect of various active compounds in tea on anti-hypertension (28). Numerous reports indicate a strong relationship between intestinal flora and host health, and simple diet changes can reshape the host’s intestinal flora (29). Excessive salt intake efficiently induces high BP and severely disrupts the intestinal microecology diversity and structure (1, 4). It has been reported that different tea extracts or active compounds could alter the composition and metabolism of the gut microbiota, directly or indirectly regulating the host health through a variety of disease model validations (13). However, the regulation mechanism of tea on hypertension driven by a high-salt diet remains poorly understood. The present study was designed to determine the effect of tea on BP and its potential regulatory mechanism.

In the current study, both GT and OLT supplementation showed a suppressive effect on BP and a protective effect on cardiac and renal tissue injuries but a limited impact on body weight. A similar result also was reported by Szulińska et al., which showed that the anti-hypertensive effects of GT and OLT were not conducted by intervening in body weight (30). Our current findings are consistent with those of Xu and Tanida, who found that GT drinking for 3–16 weeks significantly reduced systolic and diastolic BP in the hypertensive subjects tested and OLT drinking for 14 weeks reduced BP elevation in spontaneously hypertensive rats (26, 31). Besides, studies have shown that a low dose of GT extract (1 mg/kg/day) could significantly improve myocardial stiffness and cardiac compliance of deoxycorticosterone acetate-salt hypertensive rats (32). Although, our results also reveal that the anti-hypertensive activity of OLT was better than that of GT. It seems possible that this result is due to their different chemical compositions (Table 1). Owing to the distinctions in the category and source of tea, the distribution of active compounds for lowering BP is significantly different.

In addition to BP, GT or OLT intake also significantly reduced the mRNA expression of ACE and ET-1 and greatly increased the mRNA expression of eNOS. Ang II and ALD levels were significantly decreased for serum BP regulators, and NO level was markedly increased with the administration of OLT. Accordingly, GT supplementation remarkably reduced Ang II level and noticeably elevated NO status but had a limited effect on the ALD level. Renin plays a pivotal role in the development of hypertension. ACE is an integral part of the renin-angiotensin system (RAS) system, which can catalyze angiotensin I (Ang I) into Ang II with high-strength vasoconstriction activity, thereby inducing hypertension (31). Ang II can activate nicotinamide adenine dinucleotide phosphate (NADPH) to increase vascular superoxide anion, and the change of vascular superoxide anion plays a pivotal role in the occurrence of hypertension (12). Furthermore, Ang II can constrict blood vessels and promote the secretion of ALD. The high content of ALD will increase the content of Na^+^ in the blood, which will overload the blood volume and cause high BP (21). Previous reports revealed that anti-hypertensive candidates could prevent BP elevation, oxidative stress and inflammation (33). As expected, treatment with GT and OLT (no significance between them) significantly enhanced the enzyme activities of SOD and GPX, and significantly reduced the levels of MDA and Cre. Still, no remarkable difference in CRP level was observed. There are similarities between our results and those described by Antonello, who found that GT (6 mg/mL) could inhibit the increase of BP and oxidative stress in male SD rats caused by excessive Ang II (34). Moreover, GT supplementation (4 g/kg diet, 42 d) significantly reduced the concentration of TNF-α (a critical pro-inflammatory cytokine) in the serum of NaCl-induced hypertensive rats and enhanced the body’s total antioxidant status (30). ET-1 and eNOS are essential members of the renal endothelial function system. ET-1 is the most effective vasoconstrictor produced by the blood vessel wall and is involved in the pathogenesis of salt-sensitive hypertension in animals and humans (35). eNOS is a specific enzyme that oxidizes L-arginine to produce L-citrulline and NO. Studies have shown that the knockout of the eNOS gene in mice would produce vascular endothelial dysfunction and prone to hypertension. After transfection the eNOS gene, the damaged blood vessels can be recovered (36). Our results indicate that excessive salt intake enhanced the oxidative stress state of rats and led to an increase in the expression of ET-1, a decrease in the expression of eNOS, and an increase in the levels of Ang II and ALD. There are several possible explanations for this result. On the one hand, oxidative stress and pro-inflammatory factors can enhance ET-1 expression (12). On the other hand, ET-1 can activate the RAS system, promote the synthesis of Ang II, and release ALD (12). Our results corroborate the findings of a great deal of the previous work on tea and its active compounds in regulating BP. It is reported that GT (6 mg/mL) extracts significantly reduced the systolic and diastolic BP and oxidative stress of SD rats by inhibiting the increase in Ang II levels (34). In addition, black tea extracts exposed to porcine aortic endothelial cells could significantly boost the bioactivity of NO in aortic endothelial cells (37). GT extract regulated vascular homeostasis by its influence on the production of vasoconstrictive substances including Ang II, ET-1 as well as vasodilating substances (38). Interestingly, we found that the regulation effect of OLT was better than GT, which might be explained in part by higher EGCG and theanine contents in OLT. EGCG and theanine are considered to be the main components of tea to lower BP (12). Furthermore, it is speculated that the lower caffeine content might also be the reason for the high activity showed by OLT (39), and the stimulatory implications of caffeine could be decreased by the amount of EGCG in tea (12).

Long-term dietary salt-induced metabolic disorders contribute to aberrant intestinal microbiota *via* poorly understood mechanisms and further lead to high BP, accompanied by symptoms such as inflammation, gastrointestinal diseases, and endocrine disorders (25). The lower richness and diversity of gut microbes are observed in hypertensive individuals induced by a high-salt diet (40). Moreover, a strong relationship between microbial diversity and hypertension-related features has been reported (9). In line with these reports, our results (Figures 3A–D) show that the richness and diversity (including α and β) of the intestinal flora of hypertensive rats were significantly reduced. At the same time, GT and OLT supplementation significantly restored microbial richness and diversity, especially OLT. In addition to GT and OLT, many other candidates like yellow, white and dark teas can also increase the richness and diversity of the gut microbiota in dextran sulfate sodium-induced colitis mice (41). All these indicate the effectiveness of tea in regulating the richness and diversity of intestinal microbes, which could be attributed to the rich compounds in tea. At the phylum level, our current study indicates that a high-salt diet elevated gut colonization by bacteria of the phylum Firmicutes, with a resultant increase in the Firmicutes/Bacteroidetes ratio, which also accords with other previous studies (9). Furthermore, it is reported that the Firmicutes/Bacteroidetes ratio is increased in experimental animal and human subjects with obesity and metabolic syndrome (42).

Among members of the Firmicutes phylum, high-salt-enriched bacteria mainly belong to the *Lactobacillus* genus, belonging to the Lactobacillaceae family. It is well known that the Lactobacillaceae family and *Lactobacillus* genus, recognized intestinal probiotics, are beneficial intestinal flora for improvement of intestinal health and exhibit anti-inflammation, antidiabetic, and anti-obesity (43, 44). Recently and more strikingly, studies have shown that higher levels of *Lactobacillus* were observed in hypertensive rats (11). Moreover, it is reported that the high abundance of *Lactobacillus* is positively correlated with obesity-related features (45). Research shows that the *Lactobacillus* genus contains more than 170 species (46). A recent study showed that some Lactobacillus species like *Lactobacillus reuteri* were related to metabolic disorders with obesity (47). Thus, a high abundance of Lactobacillaceae and *Lactobacillus* may also boost the risks of hypertension induced by a high-salt diet. Early work has shown that *Enterococcus* is a natural inhabitant of the intestinal tract in humans and many animals and is a probiotic because it stimulates immunity, anti-inflammatory activity, and the hypocholesterolemic effect. It can be used as a starter in food fermentation (48). Our results show that GT treatment could significantly enrich the *Enterococcus* genus, thereby helping GT to prevent the increase in BP. However, there are also some reports that *Enterococcus* is an important opportunistic pathogen and can cause many infections (49). This controversial result may be related to different experimental conditions, design, and analytical methods, and it can be explained by more research on microbial regulation by GT. Compared with GT, OLT intake enriched more intestinal microbes, including *Allobaculum*, *Paraprevotella, Oscillospira*, *Bifidobacterium*, and *Ruminococcus*. *Allobaculum* and *Bifidobacterium* (recognized beneficial bacteria) play a valuable role in the body’s intestines and play an active role in promoting body health (50). Besides, studies have shown that the abundance of *Paraprevotella* is positively correlated with body strength (51). Chen et al. found that *Oscillospira* was closely related to human health because its abundance was positively correlated with gut microbial diversity and was inversely correlated with BP (52). *Ruminococcus* was reported to produce short-chain fatty acids (SCFAs) and was beneficial to the intestinal environment, whereas SCFAs are generally considered to have a variety of essential roles in maintaining human health, such as lowering BP, reducing inflammation, and protecting the intestinal mucosal barrier (53). In addition, our results reveal that OLT supplementation also significantly increased the abundance of *Unspecified_S24_7*, *Unspecified_Clostridiales*, and *Unspecified_Ruminococcaceae* genera. Thereinto, the Ruminococcaceae family is negatively correlated with arterial stiffness (54). However, there is limited information about *Unspecified_S24_7* and *Unspecified_Clostridiales*, and the relationship between their levels and gut health needs to be further studied. Our results further confirmed the regulatory effect of tea on the intestinal flora, which may be a critical factor in preventing the increase in BP evoked by a high-salt diet.

The abundance analysis of the top 50 OTUs further supports the above analysis, and 24 and 34 different OTUs were markedly altered by GT and OLT administrations, respectively. Therein, *Bacteroides*, *Aggregatibacter*, *Anaerofustis*, *Agrobacterium*, *Elusimicrobium*, *Prevotella*, *Sutterella*, *Paraprevotella*, *Coprococcus*, *Ruminococcus*, and *Akkermansia* were negatively and significantly correlated with hypertension. These bacteria may be involved in intestinal metabolism and microenvironment remodeling, thus exerting their indirect effects on regulating BP. For example, a recent study revealed that *Bacteroides* could play a protective role in hypertension and heart failure in hypertensive rodents (55). In addition, some bacteria, including *Faecalibacterium*, *Shuttleworthia*, *Clostridium*, *Dorea*, and *Enterobacter* genera, may also help lower BP. For instance, studies have shown that fecal *Faecalibacterium* abundance in patients with hypertension was lower than in healthy controls (55). Therefore, our results show that bacterial genera related to BP or its metabolic disorders may be potential therapeutic targets for preventing hypertension.

To further explore the implication of intestinal microbiota on BP, the known pathways related to BP regulation in the KEGG L3 pathway were explored, including histidine metabolism, phenylalanine, tyrosine, and tryptophan biosynthesis, primary bile acid biosynthesis, and secondary bile acid biosynthesis. It has been proven that L-histidine can exert anti-hypertensive effects in hypertensive models through central histamine H3 receptors (56). Additionally, the downstream metabolites of tryptophan in the intestine, including serotonin and indole, play an essential role in BP regulation (57). It has been reported that the primary receptor TGR5 was expressed on multiple tissues involved in BP regulation, and TGR5 agonism increased the eNOS activity of endothelial cells, which was beneficial to lower BP (58). Besides, intravenous injection of secondary bile acids could reduce BP in hypertensive rat models (59). As expected, GT and OLT supplementation altered these microbial metabolic pathways, and OLT exhibited a better regulation effect. These results strongly confirm that tea can alleviate high-salt-induced hypertension by regulating the metabolism of intestinal microbes.

In conclusion, both GT and OLT suppressed endothelial dysfunction and alleviated the increase in BP, oxidative stress, inflammation, and tissue damage in mice fed a high-salt diet. In addition, the disturbance of intestinal flora induced by a high-salt diet could be modulated by GT and OLT, which may be related to their differentiated composition. In particular, OLT has shown better anti-hypertension and regulation effects on intestinal flora structure and metabolism.

## Data Availability Statement

The datasets of intestinal flora of rats presented in this study can be found in online repositories. The names of the repository/repositories and accession number(s) can be found below: NCBI (accession: PRJNA811498).

## Ethics Statement

The animal study was reviewed and approved by Experimental Animal Ethics and Use Committee of Shanghai Jiao Tong University.

## Author Contributions

XY, XT, and FL: writing—original draft. JZ: data curation and validation. MW: data curation and software. XW: supervision and project administration. YW: supervision, project administration, and funding acquisition. All authors contributed to the article and approved the submitted version.

## Conflict of Interest

The authors declare that the research was conducted in the absence of any commercial or financial relationships that could be construed as a potential conflict of interest.

## Publisher’s Note

All claims expressed in this article are solely those of the authors and do not necessarily represent those of their affiliated organizations, or those of the publisher, the editors and the reviewers. Any product that may be evaluated in this article, or claim that may be made by its manufacturer, is not guaranteed or endorsed by the publisher.
