# Supplementary material for: Green and Oolong Tea Extracts With Different Phytochemical Compositions Prevent Hypertension and Modulate the Intestinal Flora in a High-Salt Diet Fed Wistar Rats
Source: Front Nutr. 2022 May 6;9:892801. doi: 10.3389/fnut.2022.892801 (PMC9121855; doi:10.3389/fnut.2022.892801)
Supplement: Supplementary file 1 [file Data_Sheet_1.doc]

**Supporting Information 1**

- 1. **DNA extraction and PCR amplification**

Total genome DNA from samples was extracted using CTAB method. DNA concentration and purity was monitored on 1% agarose gels. According to the concentration, DNA was diluted to 1ng/µL using sterile water. 16S rRNA genes of distinct regions (16S V3-V4) were amplified used specific primer (338F(5’- ACTCCTACGGGAGGCAGCAG-3’) and 806R(5’-GGACTACHVGGGTWTCTAAT-3’)) with the barcode. All PCR reactions were carried out with 15 µL of Phusion® High-Fidelity PCR Master Mix (New England Biolabs); 2 µM of forward and reverse primers, and about 10 ng template DNA. Thermal cycling consisted of initial denaturation at 98℃ for 1 min, followed by 30 cycles of denaturation at 98℃ for 10 s, annealing at 50℃ for 30 s, and elongation at 72℃ for 30 s, finally 72℃ for 5 min.

The same volume of 1XTAE buffer and PCR products was mixed and operated electrophoresis on 2% agarose gel for detection. PCR products was mixed in equidensity ratios. Then, mixture PCR products was purified with Qiagen Gel Extraction Kit (Qiagen, Germany).

- 1. **Illumina NovaSeq sequencing**

Sequencing libraries were generated usingTruSeq® DNA PCR-Free Sample Preparation Kit (Illumina, USA) following manufacturer's recommendations and index codes were added. The library quality was assessed on the Qubit@ 2.0 Fluorometer (Thermo Scientific). At last, the library was sequenced on an Illumina NovaSeq platform and 250 bp paired-end reads were generated.

- 1. **Bioinformatics analysis**

The analysis was conducted by following the "Atacama soil microbiome tutorial" of Qiime2docs along with customized program scripts (https://docs.qiime2.org/2019.1/). Briefly, raw data FASTQ files were imported into the format which could be operated by QIIME2 system using qiime tools import program. Demultiplexed sequences from each sample were quality filtered and trimmed , de-noised, merged, and then the chimeric sequences were identified and removed using the QIIME2 data2 plugin to obtain the feature table of amplicon sequence variant (ASV). The QIIME2 feature-classifier plugin was then used to align ASV sequences to a pre-trained GREENGENES 13_8 99% database (trimmed to the V3-V4 region bound by the 338F/806R primer pair) to generate the taxonomy table. Any contaminating mitochondrial and chloroplast sequences were filtered using the QIIME2 feature-table plugin. Appropriate methods include ANCOM, ANOVA, Kruskal Wallis, LEfSe and DEseq2 were employed to identify the bacteria with different abundance among samples and groups. Diversity metrics were calculated using the core-diversity plugin within QIIME2. Feature level alpha diversity indices, such as observed OTUs, Chao1 richness estimator, Shannon diversity index, and Faith’s phylogenetics diversity index were calculated to estimate the microbial diversity within an individual sample. Beta diversity distance measurements, including Bray Curtis, unweighted UniFrac and weighted UniFrac were performed to investigate the structural variation of microbial communities across samples and then visualized via principal coordinate analysis (PCoA). Redundancy analysis (RDA) was performed to reveal the association of microbial communities in relation to environmental factors based on relative abundances of microbial species at different taxa levels using the R package “vegan”. Co-occurrence analysis was performed by calculating Spearman’s rank correlations between predominant taxa and the network plot was used to display the associations among taxa. In addition, the potential KEGG Ortholog (KO) functional profiles of microbial communities was predicted with PICRUSt. Unless specified above, parameters used in the analysis were set as default.

**Table S1** Element distribution in aqueous extracts of GT and OLT

| Element (mg/g) | GT | OLT |
| --- | --- | --- |
| K | 52.31±0.34 | 62.57±0.26^**^ |
| Ca | 0.96±0.33^a^ | 0.43±0.06^**^ |
| Mg | 3.85±0.79^c^ | 5.81±0.23^**^ |
| P | 0.02±0.01^c^ | 0.13±0.06^**^ |
| Zn | 0.37±0.01 | 0.01±0.00^**^ |
| Fe | 0.51±0.02 | 0.03±0.01^**^ |
| Cu | 1.47±0.25 | 0.62±0.11^**^ |
| Mn | 0.04±0.01^b^ | 0.12±0.01^**^ |
| Ni | ND | ND |
| Cr | 2.40±0.32 | 0.73±0.32^**^ |
| Al  Se (mg/kg) | 3.42±0.24  4.41±0.12 | 4.25±0.28^**^  ND |

Abbreviation: ND, not detected; GT, green tea; OLT, oolong tea.


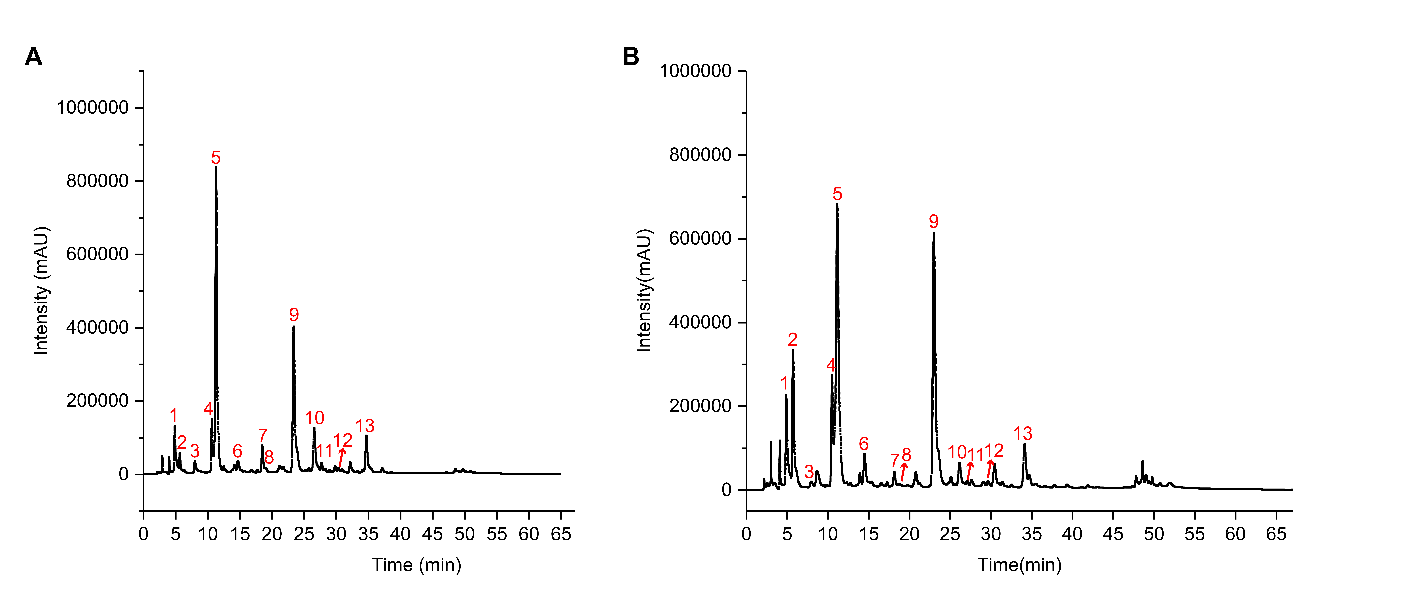


**Figure S1.** HPLC chromatograms of GT (A) and OLT (B) under 280 nm. (1) Theobromine; (2) Gallic acid; (3) GC; (4) Caffeine; (5) EGC; (6) Catechin; (7) EC; (8) Caffeic acid; (9) EGCG; (10) GCG; (11) p-Coumaric acid; (12) Ferulic acid; (13) ECG.

Abbreviation: GT, green tea; OLT, oolong tea; GC, gallocatechin; EGC, epigallocatechin; EC, epicatechin; EGCG, epigallocatechin gallate; GCG, gallocatechin gallate; ECG, epicatechin gallate.


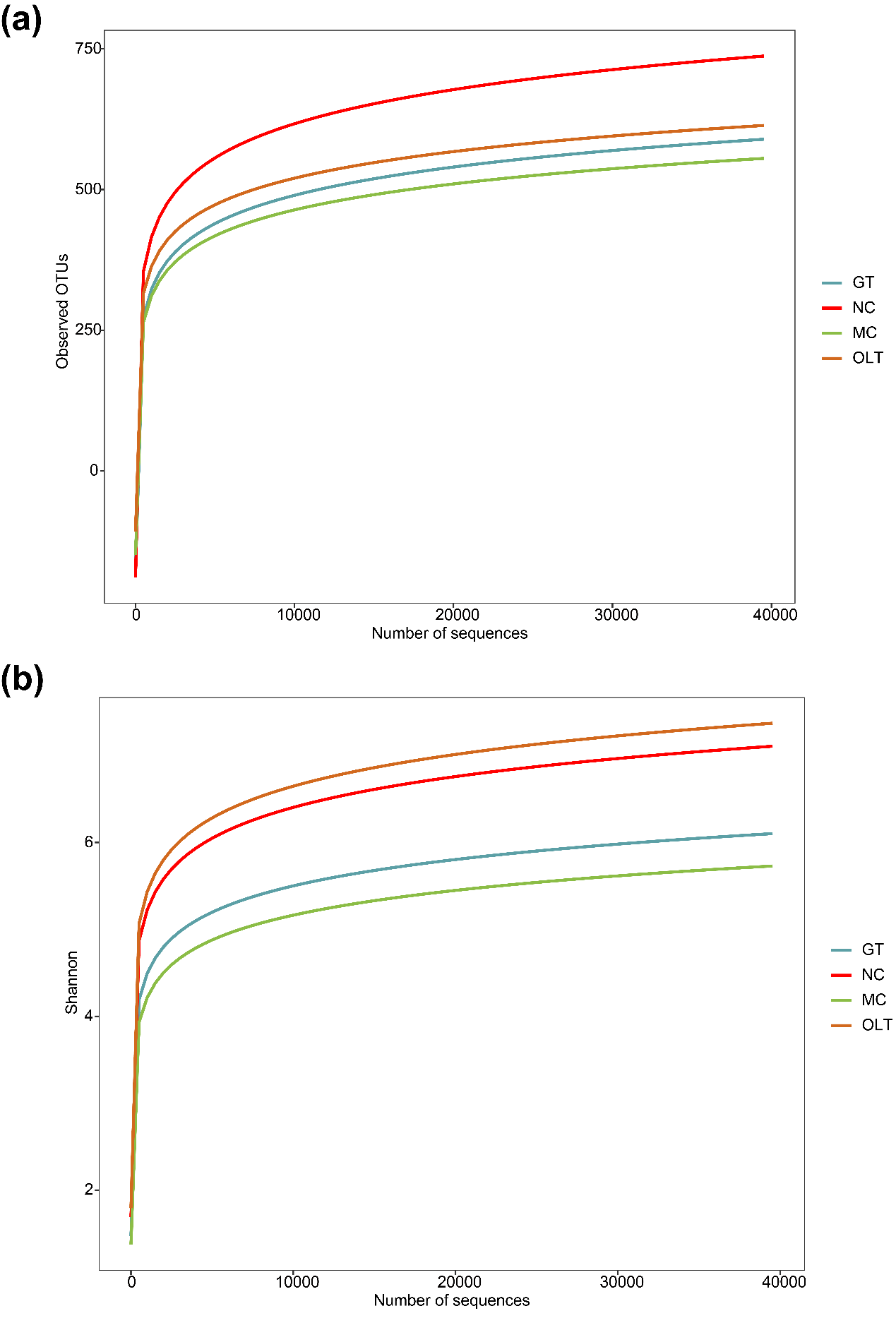


**Figure S2.** The OTU (a) and Shannon (b) rarefaction curves of fecal samples (n=6). Abbreviation: OUT, operational taxonomic unit; GT, green tea; OLT, oolong tea; MC, model control; NC, normal control.
